# Exertional fat embolism after hip joint replacement: a case report

**DOI:** 10.1186/1752-1947-8-426

**Published:** 2014-12-15

**Authors:** Rong Bing, John Yiannikas

**Affiliations:** 7 Cardiology Department, Level 3 West, Concord Repatriation General Hospital, Hospital Road, Concord, NSW 2139 Australia; 8 University of Sydney, Sydney, NSW 2006 Australia

**Keywords:** Exertional, Fat embolism, Joint replacement, Stress echocardiography

## Abstract

**Introduction:**

We present the case of a patient with exertional fat embolism on isolated exercise of his right leg two and four months after right total hip joint replacement. His immediate post-operative period had also been complicated by an acute episode of chest pain and hypotension, treated as acute coronary syndrome. To the best of our knowledge, this is the first reported case of exertional fat embolism following orthopedic surgery.

**Case presentation:**

A 71-year-old Caucasian man underwent elective cementless total right hip joint replacement. His acute post-operative period was complicated by an episode of chest pain and hypotension. This was treated as acute coronary syndrome. Two months later, a routine stress echocardiography demonstrated a shower of small, echodense bubbles in his right heart, reproduced on exercise of his right leg but not his left. Computed tomography pulmonary angiography excluded pulmonary thromboemboli. A technetium-99m colloid scan confirmed pulmonary fat emboli. Similar findings occurred again four months after the operation but had resolved at six months.

**Conclusions:**

Fat embolism is a well-described phenomenon in the acute setting after long-bone trauma or intramedullary manipulation, and the rare fat embolism syndrome can be fatal. Exertional fat embolism months after joint replacement, however, is an undescribed phenomenon that may have implications in the sub-acute post-operative phase. This may be of particular interest to those involved in orthopedics, cardiology and rehabilitation, but the large volume of patients undergoing joint replacements may broaden the clinical scope of this unusual presentation far beyond these specialties.

**Electronic supplementary material:**

The online version of this article (doi:10.1186/1752-1947-8-426) contains supplementary material, which is available to authorized users.

## Introduction

Fat embolism is a form of non-thrombotic pulmonary embolism and is a recognized complication of long-bone fractures and orthopedic surgery, which together account for up to 90% of cases [1]. Fat globules enter the venous circulation following disruption of the intramedullary cavity and the venous sinusoids within. Fat embolus syndrome is a distinct clinical entity with classical findings of an altered mental state, hypoxia and petechiae in addition to fever, tachycardia and tachypnea [2]. Lung injury is thought to be caused by mechanical alveolar obstruction as well as direct endothelial and pneumocyte damage from the release of free fatty acids. Entry to the systemic circulation may occur via an intra-cardiac shunt or transpulmonary passage across pulmonary capillaries. This syndrome is rare, with an incidence of 0.9% to 2.2% in long-bone fractures [3], and is associated with significant morbidity and mortality. Fat emboli in the absence of this clinical syndrome, however, can be readily seen during orthopedic procedures, with some series demonstrating intra-operative echocardiographic findings in up to 93% of cemented hip arthroplasties, depending on operative techniques [4]. Although it is often unclear if there is any long-term clinical consequence [5], transcranial Doppler can demonstrate intra-cerebral embolic passage, and structural brain abnormalities have been reported on magnetic resonance imaging [6, 7].

This case represents an unreported phenomenon of exertional fat embolism occurring two and four months after hip joint replacement. Whilst our patient was asymptomatic at the time of these findings, our experience with this case may suggest that exertional embolism has a causative role in patients with otherwise unexplained respiratory or neurological symptoms some months after surgery.

## Case presentation

A 71-year-old Caucasian man with osteoarthritis underwent an elective total right hip joint replacement. He had a history of ischemic heart disease with a drug-eluting stent deployed to an 80% lesion in his left anterior descending artery in 2007; the indication was stable angina. At this stage there was a residual 50% to 60% lesion in his right coronary artery. Stress echocardiography one year prior to the hip replacement was negative for ischemia at 90% of maximum predicted heart rate. Other co-morbidities included hypertension, dyslipidemia, type 2 diabetes and epilepsy.

His intra-operative course was uncomplicated. A posterior approach was taken. A 52mm acetabular component with a ceramic liner was press-fitted in place; the stem was also press-fitted and a ceramic femoral head used. Immediately after the operation, however, our patient experienced chest pain and was hypotensive with a blood pressure of 90/70mmHg with associated tachycardia and hypoxia. A cardiorespiratory examination was unremarkable and there were no neurological or dermatological changes seen at this time. Results of an electrocardiogram were normal; chest radiography showed a possible left lung base opacity. A high-sensitive troponin-T assay detected a rise in troponin T from 29ng/L to 528ng/L. Therapy for acute coronary syndrome was instituted and our patient recovered. A subsequent in-patient coronary angiography revealed a patent left anterior descending artery stent, minor left circumflex artery disease, and a 60% lesion in his right coronary artery. This was felt to be the culprit lesion and a drug-eluting stent was deployed. His subsequent post-operative recovery was unremarkable.

**Figure 1:**
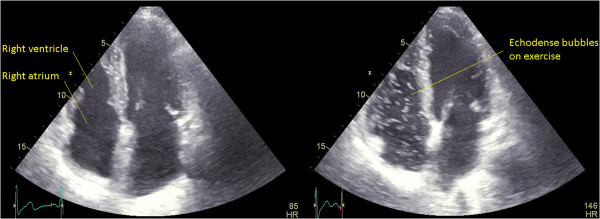
**Exertional fat embolism on treadmill stress echocardiography.** Apical four-chamber views showing no bubbles in the right heart at rest but an extensive shower of small echodense bubbles in the right heart on treadmill exercise stress testing, consistent with fat emboli. HR, heart rate.

**Figure 2:**
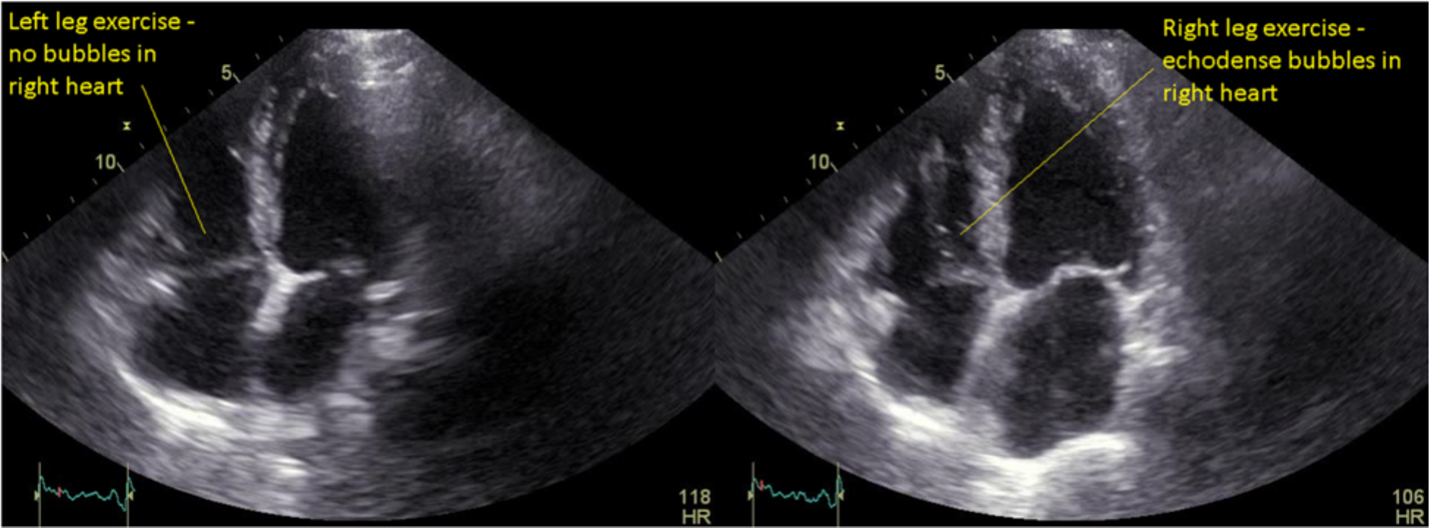
**Exertional fat embolism on bicycle stress echocardiography with isolated exercise of the right leg.** Apical four-chamber views directly following the previous study (Figure 1), showing no bubbles in the right heart on isolated left leg exercise but a small number of echodense bubbles on isolated right leg exercise. Exercise was performed with a supine bicycle and sequential use of the left and right legs. HR, heart rate.

**Figure 3:**
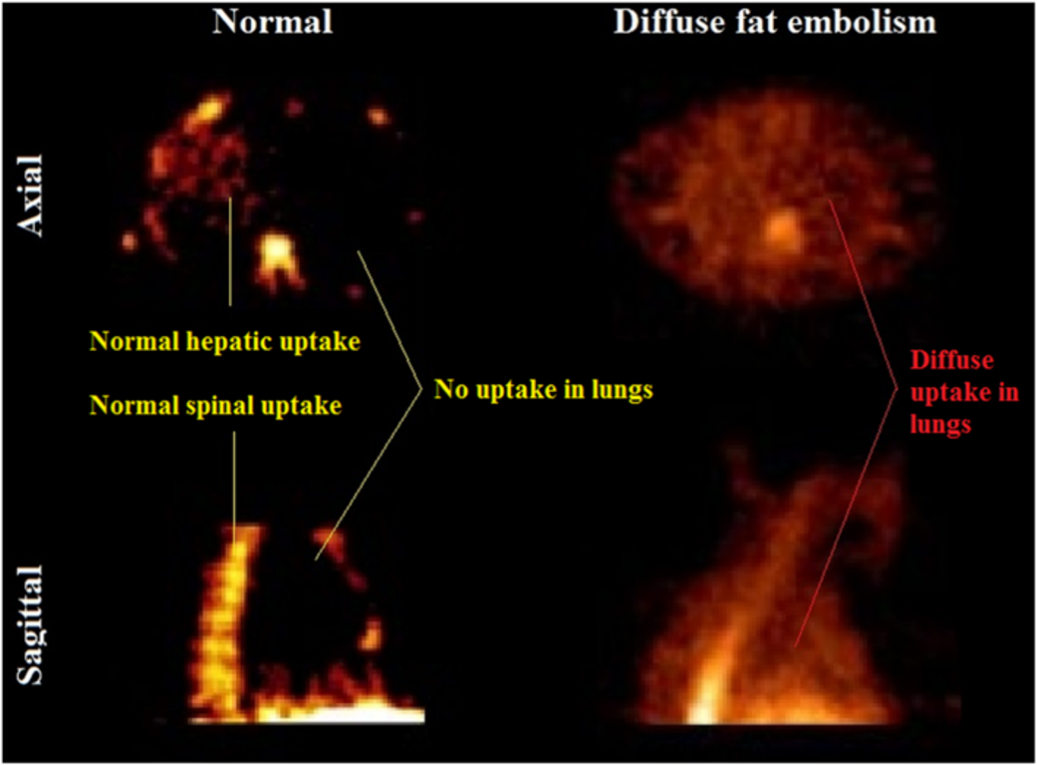
**Technetium-99m nuclear scan demonstrating diffuse fat embolism.** Axial and sagittal views. The left panel demonstrates a normal scan with physiological hepatic and spinal marrow uptake of colloid as marked. The lung fields are clear. The right panel demonstrates comparable slices from our patient’s colloid scan, showing diffuse uptake through the lungs fields as indicated, consistent with fat emboli.

Our patient attended a routine review at two months. He was asymptomatic at this time. A treadmill stress echocardiography was performed. At peak exercise, an extensive shower of small echodense bubbles in his right heart was seen, consistent with fat emboli (Additional file 1: Video 1, Figure 1). Our patient remained asymptomatic. Directly after this, repeat stress echocardiography was undertaken on a supine exercise bicycle, allowing isolated exercise of each leg. Small bubbles were reproduced with exercise of his right leg but not his left (Additional file 2: Video 2, Figure 2). A technetium-99m colloid scan [8] confirmed pulmonary fat emboli (Figure 3). Computed tomography pulmonary angiography showed no thromboemboli. Similar findings were found on stress echocardiography at four months, although diminished, with resolution at six months.

Our patient has recovered fully and has subsequently undergone elective left total hip joint replacement with a cemented prosthesis. There were no further clinical events or echocardiographic findings to suggest fat embolism after this second operation. Our patient remains well.

## Conclusions

This case demonstrates the phenomenon of an acute post-operative event, treated as primary coronary ischemia, followed by exertional non-thrombotic pulmonary emboli up to four months after hip joint replacement. Showers of echodense material seen in our patient’s right heart on echocardiography were reproduced on exercise of the affected limb, with imaging findings consistent with fat emboli. Exercise of the unaffected limb, a particularly useful maneuver in this case, did not promote the release of any echogenic material. Pulmonary fat emboli were confirmed on technetium-99m nuclear scanning following these findings. It may be postulated that the acute post-operative event was related to fat embolism, but this is a hypothesis that cannot be proven.

The possibility of lower limb venous thromboemboli cannot definitely be excluded but the appearance of small uniform particulate matter is typical of fat embolism on echocardiography, as reported by this institution previously [9]. The nuclear scan was also consistent with fat emboli. Furthermore, our patient received the standard duration of post-operative prophylaxis against venous thrombosis.

The timing of the findings noted above far exceeds the currently recognized course of post-operative fat embolism, and exertional events have not been previously reported. Although the prosthetic joint itself may be well-seated and stable, the potential disruption of the intramedullary cavity and venous sinusoids caused by the stem component may persist longer than is currently recognized, thereby allowing ongoing passage of fat globules into the venous circulation. Whilst there were no evident clinical sequelae at six months in this case, the exertional fat emboli seen here indicates the potential for a pathogenic role in patients with unexplained respiratory or neurological deterioration weeks, rather than hours to days, after orthopedic surgery.

## Consent

Written informed consent was obtained from the patient for publication of this case report and any accompanying images. A copy of the written consent is available for review by the Editor-in-Chief of this journal.

## Electronic supplementary material

Additional file 1: Video 1: MPEG4 movie file showing exertional fat emboli on stress transthoracic echocardiography (apical four chamber view) as described in the legend for Figure 1. (MP4 3 MB)

Additional file 2: Video 2: MPEG4 movie file showing exertional fat emboli on stress transthoracic echocardiography (apical four chamber view) with isolated right leg exercise, as described in the legend for Figure 2. (MP4 3 MB)
